# High density oligonucleotide array analysis of interferon-**α**2a sensitivity and transcriptional response in melanoma cells

**DOI:** 10.1054/bjoc.2001.1865

**Published:** 2001-07

**Authors:** U Certa, M Seiler, E Padovan, G C Spagnoli

**Affiliations:** Roche Genetics, F. Hoffmann-La Roche Ltd., Bau 93/610, Basel, 4070, Switzerland; Department of Surgery, Division of Research, University of Basel, Basel, Switzerland

**Keywords:** IFN-α sensitivity, melanoma, DNA microarrays, gene expression, pharmacogenomics

## Abstract

Interferon alpha (IFN-α) represents an adjuvant therapy of proven effectiveness in increasing disease-free interval and survival in subgroups of melanoma patients. Since high doses of cytokine are required, the treatment is often accompanied by toxic side effects. Furthermore, naturally occurring insensitivity to IFN-α may hamper its therapeutic efficacy. Clinical, molecular or immunological markers enabling the selection of potential responders have not been identified so far. To explore the molecular basis of IFN-α responsiveness, we analysed the expression pattern of about 7000 genes in IFN-α sensitive and resistant cell lines and we compared the transcription profiles of cells cultured in the presence or absence of the cytokine using high-density oligonucleotide arrays. Melanoma cell lines were screened for their sensitivity to proliferation inhibition and HLA class I induction upon IFN-α treatment by standard 3H-thymidine incorporation and flow-cytometry. The study of 4 sensitive and 2 resistant cell lines allowed the identification of 4 genes (RCC1, IFI16, hox2 and h19) preferentially transcribed in sensitive cells and 2 (SHB and PKC-ζ) preferentially expressed in resistant cells. IFN-α stimulation resulted in the expression of a panel of 19 known inducible genes in sensitive but not in resistant cells. Moreover a group of 30 novel IFN-α inducible genes was identified. These data may provide a useful basis to develop diagnostic tools to select potential IFN-α responders eligible for treatment, while avoiding unnecessary toxicity to non-responders. Furthermore, by extending the knowledge of the polymorphic effects of IFN-α on gene expression, they offer novel clues to the study of its pleiotropic toxicity. © 2001 Cancer Research Campaign http://www.bjcancer.com


*British Journal of Cancer* (2001) **85**(1), 107–114
© 2001 Cancer Research Campaign
doi: 10.1054/ bjoc.2001.1865, available online at http://www.idealibrary.com on IDEAL®

http://www.bjcancer.com

### High density oligonucleotide array analysis of interferon-α2a sensitivity and transcriptional response in melanoma cells

**U Certa[1], M Seiler[1], E Padovan[2] and GC Spagnoli[2]**

[1]Roche Genetics, Bau 93/610, F. Hoffmann-La Roche Ltd., 4070, Basel, Switzerland; [2]Department of Surgery, Division of Research, University of Basel, Basel, Switzerland

**Summary** Interferon alpha (IFN-α) represents an adjuvant therapy of proven effectiveness in increasing disease-free interval and survival in subgroups of melanoma patients. Since high doses of cytokine are required, the treatment is often accompanied by toxic side effects. Furthermore, naturally occurring insensitivity to IFN-α may hamper its therapeutic efficacy. Clinical, molecular or immunological markers enabling the selection of potential responders have not been identified so far. To explore the molecular basis of IFN-α responsiveness, we analysed the expression pattern of about 7000 genes in IFN-α sensitive and resistant cell lines and we compared the transcription profiles of cells cultured in the presence or absence of the cytokine using high-density oligonucleotide arrays. Melanoma cell lines were screened for their sensitivity to proliferation inhibition and HLA class I induction upon IFN-α treatment by standard 3H-thymidine incorporation and flow-cytometry. The study of 4 sensitive and 2 resistant cell lines allowed the identification of 4 genes (RCC1, IFI16, hox2 and h19) preferentially transcribed in sensitive cells and 2 (SHB and PKC-ζ) preferentially expressed in resistant cells. IFN-α stimulation resulted in the expression of a panel of 19 known inducible genes in sensitive but not in resistant cells. Moreover a group of 30 novel IFN-α inducible genes was identified. These data may provide a useful basis to develop diagnostic tools to select potential IFN-α responders eligible for treatment, while avoiding unnecessary toxicity to non-responders. Furthermore, by extending the knowledge of the polymorphic effects of IFN-α on gene expression, they offer novel clues to the study of its pleiotropic toxicity. © 2001 Cancer Research Campaign http://www.bjcancer.com

**Keywords**: IFN-α sensitivity; melanoma; DNA microarrays; gene expression; pharmacogenomics

IFN-α is widely used in the therapy of melanoma (Agarwala and Kirkwood, 1997; Grob et al, 1998). Although in subgroups of patients this treatment is of clear clinical efficacy, in others no obvious beneficial effects are observed. No clinical, immunological or molecular features predicting treatment's outcomes have been identified so far (Kirkwood, 1998).

IFN-α administration can be associated with severe toxicity, including gastrointestinal disorders, hypo- or hypertension, tachycardia, fatigue/asthenia or headache, limiting clinical applications (Vial and Descotes, 1994; Kirkwood, 1998). This wide spectrum of effects suggests pleiotropic functions of IFN-α, consistent with an extended number of target genes.

Clearly, these side effects represent a major limitation to the application of IFN-α therapy, particularly in the light of its unpredictable results.

A central element of modern pharmacogenomics is the identification of surrogate markers for drug efficacy using multiparallel approaches. The availability of tumour cell lines sensitive or resistant to well defined effects of IFN-α provides tools to search for genes whose expression is restricted to either cell type in the absence of cytokine exposure possibly leading to the development of diagnostic reagents such as antibodies or enzyme assays.

*Received 27 November 2000*
*Revised 26 March 2001*
*Accepted 29 March 2001*

*Correspondence to: GC Spagnoli*

In this work we have used high-density oligonucleotide arrays (Lipshutz et al, 1999; Rogge et al, 2000) to analyse the expression pattern of about 7000 genes in RNA samples from melanoma cell lines sensitive or resistant to IFN-α induced inhibition of proliferation and HLA class I induction. Furthermore, by applying the same technology, we have evaluated the gene expression profiles induced by IFN-α in cells sensitive or resistant to these discrete effects.

We report here on the identification of peculiar patterns of genes, of potential diagnostic relevance, preferentially expressed in either IFN-α-sensitive or -resistant melanoma cell lines. In addition, we have characterized clusters of genes whose expression can be significantly modulated by IFN-α treatment of cells, irrespective of their sensitivity to IFN-α induced inhibition of proliferation and HLA class I upregulation.

### MATERIALS AND METHODS

### Cell lines and culture conditions

ME15, ME51, ME59 and ME67 cell lines were generated in our laboratory upon culture of cell suspensions derived from surgically excised melanoma metastases (Lüscher et al, 1994). A375 cell line was a gift from Dr Eberle (Basel, Switzerland) where as D10 cell line was provided by Dr Rimoldi (Lausanne, Switzerland). All cell lines were cultured in RPMI medium supplemented with 10% FCS, glutamine (2 mM), sodium pyruvate (1 mM), non-essential aminoacids, antibiotics and HEPES buffer

(10 mM) (all from GIBCO Life Sciences, Paisley, UK). When confluent, the cells were passaged by trypsinization.

### Proliferation assays

Cell proliferation was evaluated upon culture of 5000 cells per well in flat bottom 96 wells plates (Becton Dickinson Labware, Franklin Lakes, NJ, USA) in the presence or absence of the indicated concentrations of IFN-α2a (Hoffmann-LaRoche, Basel, Switzerland) over a 5 day period. De novo DNA synthesis was measured by 3H-thymidine incorporation following overnight incubation in the presence of the tracer, according to standard methods.

### HLA class I expression

Surface expression of HLA class I was monitored by flow-cytometry, using a FITC-labelled mAb specific for a monomorphic determinant of HLA-A-B-C heavy chain or control, isotype matched reagents (Pharmingen, San Diego, CA, USA), in cells cultured for 48 hours in the presence or absence of IFN-α. Mean fluorescence intensity (MFI) of stained cells was quantitatively analysed.

### Detection of IFN-α receptor mRNA by RT-PCR

Double stranded cDNA used for the microarray experiments (lines ME15, A375, ME51, ME59, ME67 and D10, see below) was used as template for the amplification of a 370 base pair fragment of the IFNAR2 receptor (Genbank: L42243) (Lutfalla et al, 1995). Amplification was carried out using a commercial kit (Roche Molecular Biochemicals; Cat-Nr.1 939 823) and the oligonucleotides 5′-TCA TAA GGA TGA GGC TGT GAG GAG-3′ (NT 10–34) and 5′-TGT CCA GTG TCT TGG GTA ATG CAC-3′ (NT 380–366) following the manufacturers' instructions. Samples from 25 cycles PCR were subjected to agarose electrophoresis and photographed under UV transillumination.

### Oligonucleotide array analysis

Cultured melanoma cells were harvested by scraping and total cellular RNA was extracted (Mahadevappa and Warrington, 1999; Rogge et al, 2000). 10 μg from each sample were reverse transcribed, labelled and processed by using a commercial kit (Affymetrix, Santa Clara, CA) according to the supplier's instructions (Fambrough et al, 1999). Upon alkaline heat fragmentation, cDNA were hybridized to the arrays following standard procedures as supplied with the microchips (Affymetrix, Santa Clara, CA). Raw data were collected with a confocal laser scanner (Hewlett Packard, Palo Alto, CA) and pixel levels were analysed using a commercial software (GeneChip v3.1, Affymetrix, Santa Clara, CA). Expression levels for each gene were calculated as normalized average difference (nAD) of fluorescence intensity as compared to hybridization to mismatched oligonucleotides, expressed in arbitrary units. Figure 1 shows examples of genes scoring positive or negative in different cell lines as detectable on the chip unit. On average, >25% of the genes under investigation were positive in the cell lines tested.

A threshold of 20 nAD units was assigned to any gene with a calculated expression level lower than 20, since mRNA levels in this low range could not be reliably assessed. Array to array variations did not exceed 2% based on the hybridization of one sample to 5 arrays from the same batch in a pilot study (Certa and Neeb unpublished). In specific experiments (see below), change factors (CF) of fluorescence levels, expressed as nAD units, related to IFN-α exposure were also calculated. In order to exclude artifacts, only genes with robust change factors, greater than 3-fold, were included in the analysis. By applying these criteria, about 50–70 genes were found to be modulated depending on the cell lines under investigation. Genes were clustered according to their mode of regulation (up = upregulated; dn = downregulated; ~ = unmodulated).

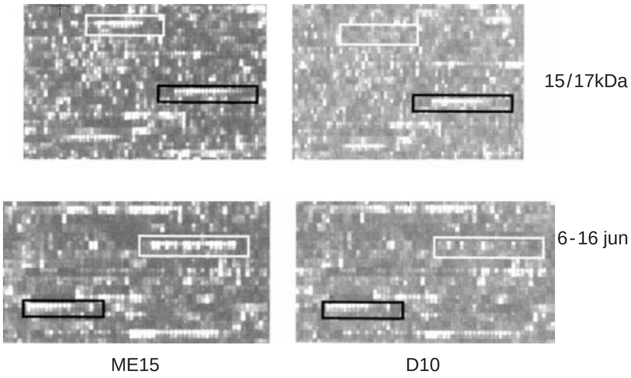

**Figure 1** Detection of gene expression by high density oligonucleotide arrays. ME15 and D10 cells were cultured for 48 hours in the presence of IFN-α (100 U ml$^{-1}$). RNA was then extracted, reverse transcribed and hybridized to high-density oligonucleotide arrays as described in the text. Positive gene expression is detectable as fluorescent signal upon cDNA hybridization on series of overlapping oligonucleotides aligned in adjacent areas. The figure reports the detection of the expression of genes encoding 15/17 kDa (upper panels) and 6–16 jun (lower panels) in ME15 but not in D10 cells (white frames). In contrast, house-keeping genes encoding ribosomal protein L39 (upper panels, Genbank no. D79205) and lactate dehydrogenase (lower panels, Genbank no. X02152) appear to be expressed in both samples (black frames)

**Table 1** Effects of IFN-α on established melanoma cell lines

| Cell line | Proliferation inhibition ($IC_{50}$)[a] | HLA class I induction[b] |
|---|---|---|
| A375 | + (100 U ml$^{-1}$) | (442 vs. 305) |
| D10 | – | (184 vs. 196) |
| ME15 | + (100 U ml$^{-1}$) | (521 vs. 257) |
| ME51 | + (10 U ml$^{-1}$) | (980 vs. 135) |
| ME59 | + (10 U ml$^{-1}$) | (559 vs. 380) |
| ME67 | – | (175 vs. 203) |

[a]Melanoma cell lines were cultured in the presence of IFN-α concentrations ranging between 1 and 1000 U ml$^{-1}$. 3H-thymidine incorporation was measured daily over a 5 day culture period following an 18 hour pulsing time. $IC_{50}$ is the IFN-α concentration inducing at least a 50% inhibition of the maximal proliferative activity detectable in individual experiments.
[b]Melanoma cells were stained with HLA class I specific monomorphic mAbs following a 2 day culture in the presence (left digits) or absence (right digits) of IFN-α (100 U ml$^{-1}$) and tested by flow-cytometry. Data are expressed as mean fluorescence intensity of labelled cells.

 *© 2001 Cancer Research Campaign*

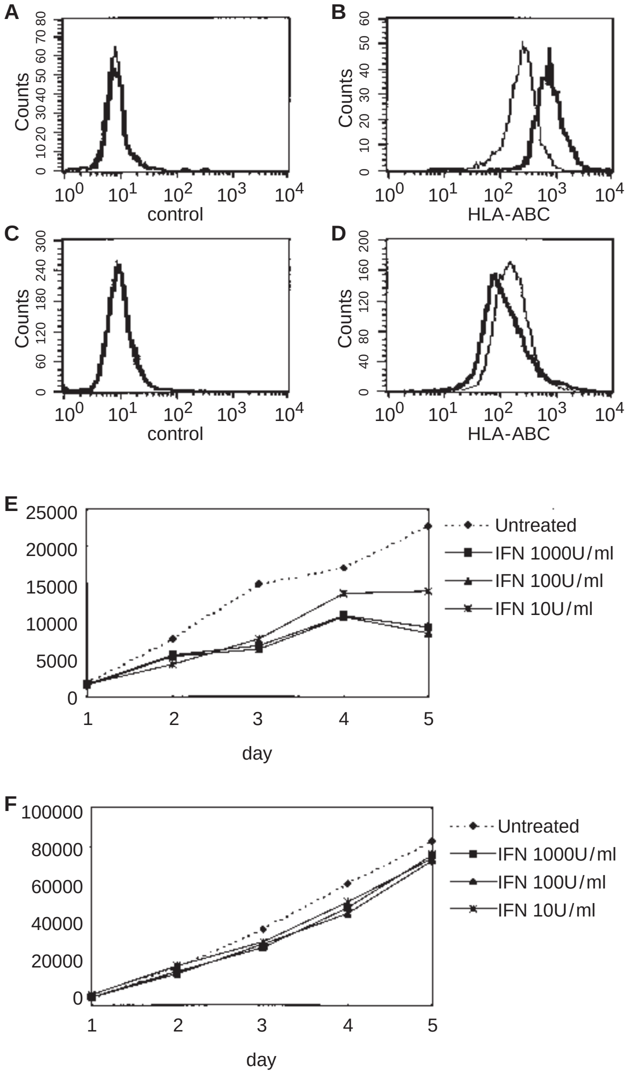

**Figure 2** IFN-α induced inhibition of proliferation and HLA class I upregulation in melanoma cell lines. ME15 (panels **A** and **B**) and D10 (panels **C** and **D**) melanoma cell lines were cultured in the presence (empty histograms) or in the absence (shaded histograms) of IFN-α (100 U m$^{-1}$) for 48 hours. Cells were then stained with a mAb recognizing a monomorphic HLA-A-B-C determinant (panels **B** and **D**) or an isotype matched control reagent (panels **A** and **C**). The proliferative capacity of the 2 cell lines (ME15, panel **E** and D10, panel **F**) was also studied by 3H-thymidine incorporation over a 5 days culture period in the absence of IFN-α or in the presence of the indicated concentrations of the cytokine. Data are reported as cpm. Standard deviations, never exceeding 10% of the reported values were omitted

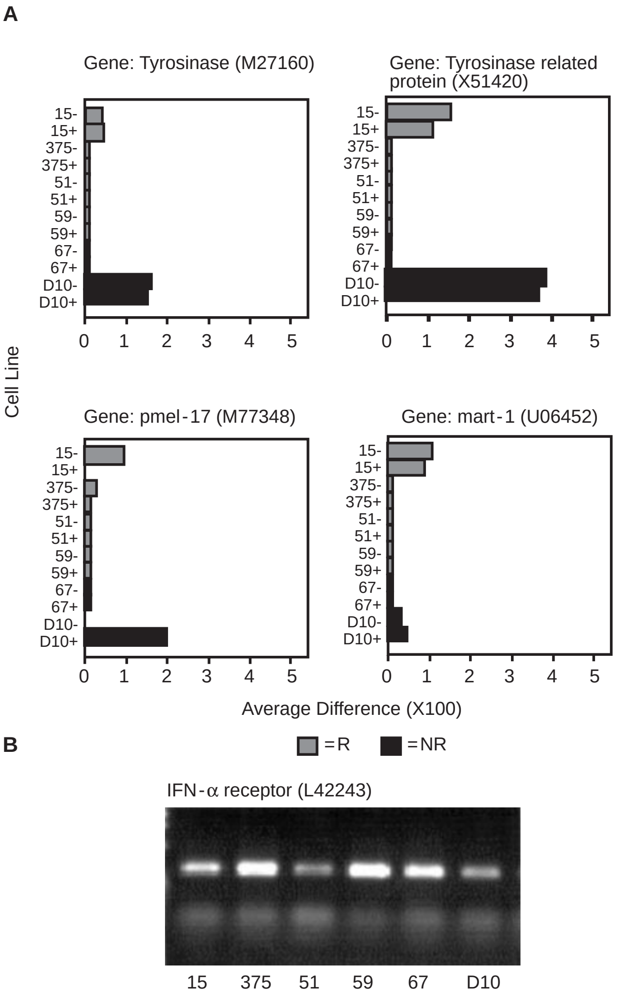

**Figure 3** Panel **A**: Expression of genes encoding tumour-associated antigens in melanoma cell lines. ME15, A375, ME51, ME59, ME67 and D10 cell lines were cultured for 48 hours in the presence (+) or absence (–) of 100 U ml$^{-1}$ IFN-α. The expression patterns of genes encoding tyrosinase, tyrosinase related protein-2, pmel-17 and mart-1 HLA restricted, tumour-associated antigens are reported. Grey bars refer to IFN-α sensitive cell lines and black bars refer to IFN-α resistant lines (see Table 1). Data are presented as normalized average difference (nAD) of fluorescence intensity between matched and mismatched oligonucleotide probe sets, expressed in arbitrary units. Panel **B**: Detection of IFN-α receptor gene transcripts in sensitive and resistant lines by 25 cycles RT-PCR. For any other detail see ‘Materials and methods’

### RESULTS

### Identification of IFN-α sensitive and insensitive melanoma cell lines

A number of established melanoma cell lines were assayed for their sensitivity to IFN-α by testing the capacity of this cytokine to inhibit their proliferation and to increase their surface expression of HLA class I determinants. 2 cell lines (D10 and ME67) were found to be insensitive to the antiproliferative effects of IFN-α. Proliferation of ME 51 and ME59 could be at least 50% inhibited by IFN-α concentrations as low as 10 U ml$^{-1}$, whereas A375 and ME15 required a 10 times higher dose for the elicitations of similar effects (Table 1). The upregulation of HLA class I expression by IFN-α closely matched its antiproliferative effects and in no case a dissociation of the 2 activities could be observed (Table 1). Figure 2 reports representative results related to D10 and ME15 cell lines.

### Experimental set up and detection of genes encoding tumour-associated antigens in melanoma cell lines

Total cellular RNA was extracted from the sensitive and resistant melanoma cell lines characterized above, reverse transcribed and processed for hybridization to an oligonucleotide array (Hu6800FL, PN 900183, Affymetrix, Santa Clara, CA) containing probe sets from full length human genes.

**Table 2** IFN-α modulated genes

| | | D10 | D10+IFN | ME15 | ME15+IFN | CF D10 | CF ME15 | Cluster | |
|---|---|---|---|---|---|---|---|---|---|
| | | | | | | | | D10 | ME15 |
| **Cluster 1** | | | | | | | | | |
| U51127 | interferon regulatory factor 5 | 262 | 91 | 20 | 116 | –2.88 | 5.8 | ~ | up |
| X07730 | prostate specific antigen | 234 | 85 | 20 | 132 | –2.75 | 6.6 | ~ | up |
| X57522 | ring4 cdna | 143 | 49 | 20 | 97 | –2.92 | 4.85 | ~ | up |
| Z56281 | interferon regulatory factor 3 | 93 | 31 | 20 | 136 | –3 | 6.8 | ~ | up |
| J04164 | interferon-inducible protein 27-sep | 20 | 20 | 20 | 2066 | 0 | 103.3 | ~ | up |
| U50648 | interferon-inducible rna-dependent protein kinase (pkr) | 106 | 46 | 20 | 71 | –2.3 | 3.55 | ~ | up |
| X67325 | p27 | 20 | 20 | 20 | 410 | 0 | 20.5 | ~ | up |
| HG658 | major histocompatibility complex, class Ic | 1183 | 455 | 156 | 1692 | –2.6 | 10.85 | ~ | up |
| M13755 | interferon-induced 17-kda/15-kda protein | 20 | 31 | 20 | 716 | 1.55 | 35.8 | ~ | up |
| D28137 | bst-2, | 37 | 88 | 28 | 305 | 2.38 | 10.89 | ~ | up |
| M19650 | 2′, 3′-cyclic nucleotide 3prime-phosphodiesterase | 137 | 99 | 55 | 269 | 1.38 | 4.89 | ~ | up |
| X96401 | rox protein | 229 | 83 | 34 | 143 | 2.76 | 4.21 | ~ | up |
| U22970 | 16-jun gene, interferon-inducible peptide (6–16) | 20 | 24 | 43 | 2363 | 1.2 | 54.95 | ~ | up |
| X57351 | 1-8d gene from interferon-inducible gene family | 2469 | 2686 | 24 | 297 | 1.09 | 12.38 | ~ | up |
| X02874 | 2-5A synthetase (1.6 kb) | 57 | 20 | 20 | 486 | 2.85 | 24.3 | ~ | up |
| J00105 | beta-2 microglobulin gene | 1668 | 2101 | 248 | 1453 | 1.26 | 5.86 | ~ | up |
| M94880 | mhc class i (hla-a*8001) | 551 | 421 | 202 | 932 | 1.31 | 4.61 | ~ | up |
| HG2917 | major histocompatibility complex, class i, e | 353 | 322 | 147 | 813 | 1.1 | 5.53 | ~ | up |
| D49824 | hla-b null allele | 527 | 267 | 144 | 925 | 1.97 | 6.42 | ~ | up |
| **Cluster 2** | | | | | | | | | |
| M98343 | amplaxin (ems1) | 20 | 63 | 20 | 141 | 3.15 | 7.05 | up | up |
| **Cluster 3** | | | | | | | | | |
| M92642 | alpha-1 type xvi collagen (col16a1) | 20 | 115 | 20 | 20 | 5.75 | 0 | up | ~ |
| J03909 | gamma-interferon-inducible protein (ip-30) | 42 | 142 | 20 | 57 | 3.38 | 2.85 | up | ~ |
| U41515 | dss1 | 20 | 123 | 104 | 51 | 6.15 | –2.04 | up | ~ |
| J04182 | lysosomal membrane glycoprotein-1 (lamp 1) | 66 | 484 | 168 | 452 | 7.33 | 2.69 | up | ~ |
| **Cluster 4** | | | | | | | | | |
| L40387 | thyroid receptor interactor (trip 14) | 104 | 20 | 20 | 85 | –5.2 | 4.25 | dn | up |
| U72882 | interferon-induced leucine zipper protein (ifp35) | 125 | 27 | 20 | 502 | –4.63 | 25.1 | dn | up |
| U53830 | interferon regulatory factor 7a | 103 | 20 | 20 | 237 | –5.15 | 11.85 | dn | up |
| M97935 | transcription factor isgf-3 | 331 | 92 | 20 | 296 | –3.6 | 14.8 | dn | up |
| U01824 | glutamate/aspartate transporter | 339 | 35 | 20 | 91 | –9.69 | 4.55 | dn | up |
| **Cluster 5** | | | | | | | | | |
| J00212 | leukocyte interferon (ifn-alpha) | 106 | 20 | 138 | 49 | –5.3 | –2.82 | dn | ~ |
| U52513 | rig-g, | 289 | 20 | 49 | 96 | –14.45 | 1.96 | dn | ~ |
| X90846 | mixed lineage kinase 2 | 357 | 60 | 425 | 173 | –5.95 | –2.46 | dn | ~ |
| L42243 | ifnar2 gene (interferon receptor) | 62 | 20 | 46 | 20 | –3.1 | –2.3 | dn | ~ |
| M79462 | pml-1 | 201 | 20 | 43 | 20 | –10.05 | –2.15 | dn | ~ |
| K01900 | lymphocyte interferon alpha type 201 | 156 | 20 | 55 | 85 | –7.8 | 1.55 | dn | ~ |
| **Cluster 6** | | | | | | | | | |
| M30818 | interferon-induced cellular resistance mediator protein | 20 | 20 | 756 | 155 | 0 | –4.88 | ~ | dn |
| X15949 | interferon regulatory factor-2 (irf-2) | 20 | 20 | 67 | 20 | 0 | –3.35 | ~ | dn |

D10 and ME15 cell lines cultured for 48 hours in the absence (D10 and ME15, respectively) or in the presence of 100 U $ml^{-1}$ IFN-α (D10 +IFN and ME15+IFN, respectively) were used to assay cytokine modulated gene expression as matched with data obtained in fibrosarcoma cells (Der et al, 1998). Expression levels for each gene were calculated as normalized average difference (nAD) of fluorescence intensity as compared to hybridization to mismatched oligonucleotides, expressed in arbitrary units. A threshold of 20 nAD units was assigned to any gene with a calculated expression level lower than 20, since mRNA levels in this low range could not be reliably assessed. Genes displaying modulations (change factor = CF) greater than 3-fold were included in the analysis and they were clustered according to their mode of regulation (up = upregulated; dn = downregulated; ~= unmodulated). This analysis yielded 6 clusters of genes. Cluster 1 contains genes only upregulated in the IFN-α sensitive ME15 line. Cluster 2 includes amplaxin, upregulated in both lines and cluster 3 comprises genes only upregulated in the D10 resistant line. Cluster 4 refers to genes downregulated in D10 and upregulated in ME15 cells. Cluster 5 includes genes downregulated in D10 and cluster 6 refers to genes downregulated in ME15 cells.

© 2001 Cancer Research Campaign

**Table 3** Novel IFN-α inducible genes

| | | D10 | D10+IFN | ME15 | ME15+IFN | CF D10 | CF ME15 | Cluster | |
|---|---|---|---|---|---|---|---|---|---|
| **Cluster 1** | | | | | | | | | |
| L37043 | casein kinase i epsilon | 218 | 221 | 20 | 441 | 2.01 | 22.05 | ~ | up |
| D32050 | alanyl-trna synthetase | 1458 | 761 | 21 | 427 | –1.92 | 20.33 | ~ | up |
| D28137 | bst-2 | 37 | 88 | 28 | 305 | 2.38 | 10.89 | ~ | up |
| S81914 | iex-1=radiation-inducible immediate-early gene | 20 | 21 | 31 | 312 | 1.05 | 10.06 | ~ | up |
| X95325 | dna binding protein a variant | 634 | 253 | 58 | 375 | 2.51 | 6.47 | ~ | up |
| U91316 | acyl-coa thioester hydrolase | 253 | 428 | 141 | 706 | 1.69 | 5.01 | ~ | up |
| U47025 | fetal brain glycogen phosphorylase b | 672 | 465 | 193 | 924 | –1.45 | 4.79 | ~ | up |
| Z26491 | gene catechol o-methyltransferase | 238 | 181 | 73 | 337 | –1.31 | 4.62 | ~ | up |
| L13210 | mac-2 binding protein | 2555 | 2092 | 444 | 2016 | –1.22 | 4.54 | ~ | up |
| K03515 | neuroleukin | 2998 | 2552 | 487 | 2126 | –1.17 | 4.37 | ~ | up |
| U09579 | melanoma differentiation associated (mda-6) | 20 | 20 | 83 | 361 | 0 | 4.35 | ~ | up |
| J04444 | cytochrome c-1 gene | 886 | 2268 | 309 | 1286 | 2.56 | 4.16 | ~ | up |
| U72206 | guanine nucleotide regulatory factor (lfp40) | 238 | 162 | 113 | 459 | –1.47 | 4.06 | ~ | up |
| X76538 | mpv17 | 132 | 101 | 77 | 313 | –1.31 | 4.06 | ~ | up |
| U18009 | chromosome 17q21 clone lf113 | 674 | 423 | 187 | 735 | –1.59 | 3.93 | ~ | up |
| U69126 | fuse binding protein 2 (fbp2) | 114 | 159 | 77 | 302 | 1.39 | 3.92 | ~ | up |
| U50327 | protein kinase c substrate 80k-h gene (prkcsh) | 401 | 236 | 80 | 312 | –1.7 | 3.9 | ~ | up |
| D21235 | hhr23a protein | 388 | 346 | 150 | 582 | –1.12 | 3.88 | ~ | up |
| HG1612 | macmarcks | 588 | 505 | 401 | 1440 | –1.16 | 3.59 | ~ | up |
| X04412 | plasma gelsolin | 220 | 220 | 107 | 372 | 0 | 3.48 | ~ | up |
| U65579 | mitochondrial nadh dehydrogenase-ubiquinone | 630 | 621 | 110 | 377 | –1.01 | 3.43 | ~ | up |
| Y00264 | amyloid a4 precursor | 392 | 427 | 89 | 304 | 1.09 | 3.42 | ~ | up |
| M31013 | nonmuscle myosin heavy chain (nmhc) | 377 | 263 | 132 | 441 | –1.43 | 3.34 | ~ | up |
| M34338 | spermidine synthase | 472 | 277 | 429 | 1413 | –1.7 | 3.29 | ~ | up |
| D50914 | EST | 234 | 78 | 110 | 359 | –3 | 3.26 | ~ | up |
| U18018 | e1a enhancer binding protein (e1a-f) | 20 | 23 | 107 | 340 | 1.15 | 3.18 | ~ | up |
| U61263 | acetolactate synthase homolog | 410 | 301 | 150 | 464 | –1.36 | 3.09 | ~ | up |
| U65932 | extracellular matrix protein 1 (ecm1) | 2498 | 1066 | 300 | 907 | –2.34 | 3.02 | ~ | up |
| **Cluster 2** | | | | | | | | | |
| L35249 | vaculolar h+-atpase mr 56 000 subunit (ho57) | 20 | 357 | 92 | 227 | 17.85 | 2.47 | up | ~ |
| U70063 | human acid ceramidase | 115 | 400 | 86 | 221 | 3.48 | 2.57 | up | ~ |

D10 and ME15 cell lines cultured for 48 hours in the absence (D10 and ME15, respectively) or in the presence of 100 U $ml^{-1}$ IFN-α (D10+IFN and ME15+IFN, respectively) were used to identify novel cytokine induced genes whose expression was not found to be modulated in fibrosarcoma cells (Der et al, 1998). Expression levels for each gene were calculated as normalized average difference (nAD) of fluorescence intensity as compared to hybridization to mismatched oligonucleotides, expressed in arbitrary units. A threshold of 20 nAD units was assigned to any gene with a calculated expression level lower than 20, since mRNA levels in this low range could not be reliably assessed. Genes displaying modulations (change factor = CF) greater than 3-fold were included in the analysis and they were clustered according to their mode of regulation (up = upregulated; dn = downregulated; ~ = unmodulated). Cluster 1 includes genes inducible in ME15 sensitive but not in D10 resistant cell line. Cluster 2 comprises genes prevailingly inducible in resistant cells.

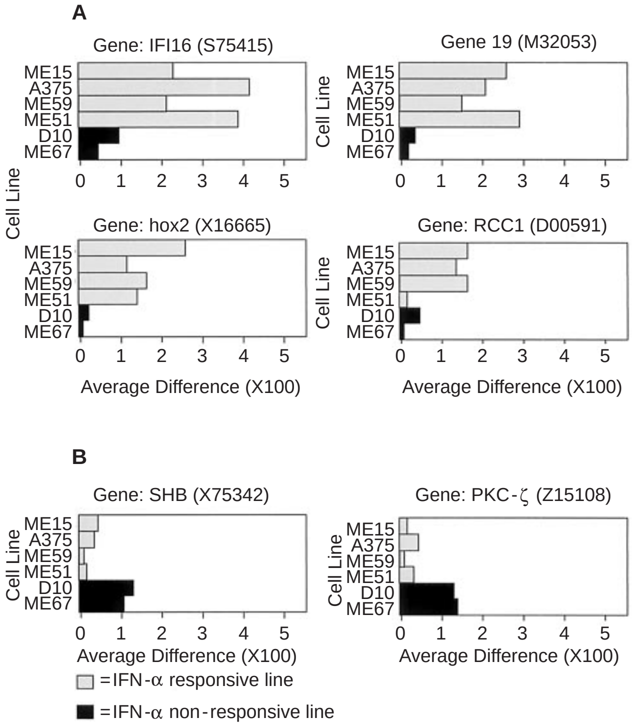

**Figure 4** Genes preferentially expressed in IFN-α sensitive (panel **A**) or resistant (panel **B**) melanoma cell lines. Oligonucleotide array expression data were collected from untreated cells. Data from the sensitive (ME15, A375, ME59, ME51, grey bars) or resistant lines (D10, ME67, black bars) were combined to identify genes preferentially expressed in either group. Data are presented as normalized average difference (nAD) of fluorescence intensity between matched and mismatched oligonucleotide probe sets, expressed in arbitrary units

We first analysed data sets for genes encoding MART-1/Melan-A, pmel-17 (gp100), TRP-2 and tyrosinase tumour-associated antigens (TAA). In agreement with concomitantly performed conventional and quantitative real time PCR assays (data not shown), the 4 genes were found to be expressed in ME15 and D10 cell lines whereas virtually no expression was detectable in A375, ME51, ME59 and ME67 cell lines (Figure 3A). These results were consistent with our previously published data, showing that D10, HLA-A2.1-positive, melanoma cells were effectively killed by HLA-A2. 1-restricted CTL lines recognizing epitopes derived from MART-1/Melan-A, pmel-17/gp 100, tyrosinase or TRP-2 proteins (Spagnoli et al, 1995; Noppen et al, 2000). In contrast, ME59 HLA-A2.1 positive cells, that did not express the genes under investigation failed to be killed by the specific CTL (Spagnoli et al, 1995). Remarkably, IFN-α treatment did not appear to influence the expression of the genes encoding these TAA.

Thus, the application of the microarray technology to the cellular system under investigation was validated by results obtained at functional and gene expression level.

Transcripts from the IFN-α receptor gene (IFNAR2) were detected at low levels (nAD ≤ 60; see also Table 2, gene cluster 5) upon microarray hybridization of the cDNA from the cell lines under investigation. To confirm and reinforce these data, however, we evaluated IFN-α receptor gene expression by using a more sensitive RT-PCR assay (Figure 3B). Although to different extents, unrelated to the level of responsiveness to the cytokine, specific transcripts could indeed be amplified from all lines.

### Detection of potential marker genes for IFN-α responsiveness

The availability of large mRNA expression data sets from 6 human melanoma cell lines well characterized for their responsiveness to IFN-α raised the possibility of identifying genes preferentially expressed in sensitive or resistant lines in the absence of cytokine treatment. Microarray data of all genes from the responder (ME15, ME51, ME59 and A375) and non-responder (D10, ME67) cells were combined and screened for genes preferentially expressed in either group.

This analysis resulted in the identification of a group of 4 genes prevailingly detectable in IFN-α sensitive cell lines (Figure 4, panel A). 2 of them, IFI16 and RCC1 encode nuclear proteins endowed with mitotic regulation and transcriptional activation capacities, respectively (Bischoff and Ponstingl, 1991; Trapani et al, 1994). A third is the hox2 homeobox gene (Acampora et al, 1989), whereas the fourth, h19 gene, encodes an untranslated RNA, involved in the DNA methylation and genetic imprinting processes (Brannan et al, 1990). Notably, however, RCC1 gene was not expressed in one IFN-α sensitive cell line (ME51).

On the other hand, 2 genes encoding likely components of signal transduction pathways, SHB and PKC-ζ (Barbee et al, 1993; Welsh et al, 1994) appeared to be preferentially expressed in IFN-α resistant D10 and ME67 cell lines (Figure 4, panel B).

### Induction of gene expression by IFN-α in sensitive and resistant cell lines

We then investigated the pattern of genes expressed in IFN-α-sensitive and -insensitive melanoma cell lines upon culture in the presence of the cytokine. Since inhibitory effects on cell proliferation were first detectable after 72 hour cultures (see Figure 1) we chose to investigate gene expression in cells cultured for 48 hours in the presence or absence of the cytokine. ME15 and D10 cell lines were studied in detail. Our analysis focused on genes which were at least 3-fold up- or down-regulated as compared to untreated cells and displayed nAD values of at least 50 in 1 of the 4 experiments.

Table 2 reports data from 6 clusters of known IFN-α modulated genes, previously found to be regulated by cytokine treatment in one human fibrosarcoma cell line (Der et al, 1998). Indeed we found that the expression of a number of them can also be modulated by IFN-α in apparently resistant cells. Cluster 1 includes genes only inducible in the sensitive ME15 cell line. As expected the expression of these genes was not significantly affected by the treatment in D10, IFN-α-resistant, cells. This set of genes includes HLA class I genes, 2-5A synthetase, TAP-1, genes encoding a number of interferon-inducible proteins, but also p27 cyclin-dependent kinase inhibitor and ROX protein (Rasmussen et al, 1993; Nigro et al, 1998). A single gene, encoding amplaxin or ems-1, and derived from a locus, 11q13, frequently amplified in tumour cells (Shuurig, 1995) appears to be induced by IFN-α in both lines (cluster 2).

Cluster 3 genes, including ip-30, a known IFN-γ inducible gene, and dss 1 were induced in D10-resistant cell line but their expression was not significantly modified in ME15 sensitive cells. Cluster 4 includes additional genes inducible by IFN-α in ME15 which are, in contrast to cluster 1, downregulated in IFN-α resistant D10 cells. Remarkably, the transcription factor ISGF-3, of relevance for IFN-α signalling belongs to this cluster that includes other IFN-related genes. Cluster 5 comprises genes downregulated

 © 2001 Cancer Research Campaign by IFN-α treatment in resistant D10 cells, but virtually unaffected in sensitive ME15 cells. Interestingly, this cluster comprises the gene encoding IFN-α receptor. Cluster 6 includes 2 genes (irf-2 and interferon-induced cellular resistance mediator) whose expression, basically undetectable in D10 resistant cell line, is downmodulated in ME15 IFN-α sensitive cells. Notably, the IRF-2 gene product is known to bind to the promoter region of IFN type I-inducible genes and to prevent transcription (Itoh et al, 1989). Downregulation could thus promote the activation of IFN inducible genes.

### Detection of novel IFN-α inducible genes

Table 3 includes genes not previously described as IFN-α inducible, whose expression was found to be upregulated upon melanoma cells treatment. Cluster 1 comprises genes only induced in sensitive cells, whereas cluster 2 refers to genes upregulated upon IFN-α treatment of apparently insensitive melanoma cells. Some of these genes obviously belong to melanocytic (melanoma differentiation antigen, mda-6) or neuroectodermic (e.g., neuroleukin or catechol o-methyltransferase; Gurney et al, 1986; Tenhunen et al, 1994) cell lineages, while other clearly inducible genes such as those encoding, for instance, plasma gelsolin or spermidine synthase escape an evident, similar, tissue-specific classification.

### DISCUSSION

IFN-α treatment is currently the only adjuvant therapy of proven effectiveness in increasing disease-free interval and overall survival in malignant melanoma, following potentially curative surgery (Kirkwood, 1998). However, the administration of relatively high doses of cytokine appears to be required (Agarwala and Kirkwood, 1996; Grob et al, 1998; Keilholz and Eggermont, 2000), frequently resulting in severe toxic side effects, ranging between flu-like symptoms, severe neuro-hepato-toxicity and myelosuppression (Vial and Descotes, 1994). Clinical, immunological or molecular features enabling a targeted selection of patients likely to take advantage of this therapy have not been identified so far (Kirkwood, 1998). Only recently, baseline white blood cell count has been suggested as a possible prognostic factor of potential clinical relevance (de La Salmonière et al, 2000). Clearly, the development of criteria predicting the potential effectiveness of IFN-α therapy would be of high clinical relevance since it would spare unnecessary toxicity to non-responders and it would contribute to the identification of responders' subgroups.

In this work we addressed the genetic profile of melanoma cell lines classified according to their sensitivity or insensitivity to critical direct effects of IFN-α, namely the inhibition of proliferation and the upregulation of HLA class I expression.

Oligonucleotide microarray technology allows us to investigate the expression of large panels of genes and appears to be ideally suited to the analysis of relatively simple cellular systems (Marton et al, 1998; Iyer et al, 1999). In our hands its sensitivity was confirmed by preliminary studies yielding results related to the expression of melanoma-associated antigens consistent with those obtained from functional assays or conventional PCR experiments.

Resistance to the antiproliferative and HLA class I-inducing effects of IFN-α does not appear to be related to major differences in the expression of genes encoding key players of the specific signal transduction chain. STAT genes included in the array (2, 4, 5a and 5b) were found to be expressed at relatively low levels (nAD ≤ 50) in all cell lines, irrespective of IFN-α responsiveness. These results are in agreement with data obtained by using different technologies, suggesting the presence of relatively functional signal transduction in IFN-α insensitive cells (Ralph et al, 1995; Wong et al, 1997). Interestingly, similar results were also obtained by testing some of the cell lines under investigation in the current work by conventional gene and protein expression techniques (Pansky et al, 2000).

A pattern of genes preferentially expressed according to typical profiles in sensitive and resistant cells clearly emerges. Genes involved in the regulation of cell proliferation, such as IFI16, h19 and RCC1, but also hox2 (Acampora et al, 1989; Brannan et al, 1990; Bischoff and Ponstingl 1991; Trapani et al, 1994), were found to be preferentially expressed in sensitive cell lines. Intriguingly, genes encoding SHB and PKC-ζ proteins, known components of defined signal transduction pathways (Barbee et al, 1993; Welsh et al, 1994), appear to be preferentially expressed in IFN-α resistant cells (Figure 4, panels A and B). These puzzling data suggest that IFN-α resistance could result from a series of active events as opposed to a merely defective activation.

IFN-α stimulation of sensitive melanoma cells resulted in the detectable upregulation of a number of known inducible genes (Table 2, cluster 1). For instance 6–16 and 27-sep genes, both more than 20-fold upregulated in human fibrosarcoma cells (Der et al, 1998) were found to be induced in ME 15 melanoma 54-and 102-fold respectively, while neither was detectable in the IFN-α resistant line D10. Another classical interferon inducible gene, 2′–5′ oligosynthase, was upregulated in ME15 but not in D10 cells. These confirmatory results further validate the integrity of the microarray analysis in this specific experimental framework. Remarkably, the 27-sep gene product, the IFN inducible Leu-13 antigen, is implicated in growth control in several cell lines (Deblandre et al, 1995). On the other hand the genes encoding IFN-induced cellular resistance mediator protein (Aebi et al, 1989) and IFN regulatory factor-2 (Schuurig, 1995) were clearly down-regulated in the sensitive ME15 cell line (Table 2, cluster 6).

Most interestingly, in cells apparently insensitive to the inhibition of proliferation and to the HLA class I induction determined by IFN-α, a significant modulation of the expression of discrete sets of genes could nevertheless be observed upon cytokine treatment. The downregulation of a set of known interferon-related genes, including those encoding the cytokine and its specific receptor was matched by the upregulation of ip-30 and dss1 genes (Luster et al, 1988; Crackower et al, 1996) (Table 2, cluster 3). Clearly, these results are compatible with the hypothesis of 'partial' effects of IFN-α, upstream of inhibition of proliferation and HLA class I induction. Importantly, these effects, on cells conventionally classified as resistant, might also offer novel insights into IFN-α related toxicity.

A further important result is represented by the identification of a group of 30 genes whose expression appears to be upregulated by IFN-α treatment in melanoma cells (Table 3), but was not reported in cells of different histological origin (Der et al, 1989). Some of these genes are typically transcribed in neuroectodermic tissues. It is tempting to speculate that their products might play a role in exogenous IFN-α induced neurotoxicity (Adams et al, 1988) or that their upregulation by endogenously produced IFN-α might be of relevance in defined neurological syndromes. Others, however, including cytochrome c-1 gene, do not obviously pertain

© 2001 Cancer Research Campaign 

to given tissue-specific transcription patterns. Further research is warranted to clarify the role eventually played by the products of these genes in discrete aspects of the polymorphic toxic effects of IFN-α.

Taken together our data provide an extended database of potential relevance in the investigation of the molecular background of IFN-α sensitivity of melanoma cells both, in clinical tumour samples and in basic cell biology studies. Ongoing studies addressing the validation of these data at the protein level might result in the characterization of reagents of clinical interest.

### ACKNOWLEDGEMENTS

Thanks are due to Prof A Eberle (Basel, Switzerland) and Dr D Rimoldi (Lausanne, Switzerland) for providing cellular reagents. This work was partially funded by research grants from the Swiss National Fund for Scientific Research (no. 31-57′473.99 to GCS) and Krebsliga beider Basel (no. 6/00 to EP).

### REFERENCES

Acampora D, D'Esposito M, Faiella A, Pannese M, Migliaccio E, Morelli F, Stornaiuolo A, Nigro V, Simeone A and Boncinelli E (1989) The human HOX gene family. *Nucleic Acids Res* **17**: 10385–10402

Adams F, Fernandez F and Mavligit G (1988) Interferon-induced organic mental disorders associated with unsuspected pre-existing neurologic abnormalities. *J Neurooncol* **6**: 355–359

Aebi M, Faeh J, Hurt N, Samuel CE, Thomis DC, Bazzogher L, Pavlovic J, Haller O and Staheli P (1989) cDNA structures and regulation of two interferon-induced human Mx proteins. *Mol Cell Biol* **9**: 5062–5072

Agarwala SS and Kirkwood JM (1996) Interferons in melanoma. *Curr Opin Oncol* **8**: 167–174

Barbee JL, Loomis CR, Deutscher SL and Burns DJ (1993) The cDNA sequence encoding human protein kinase C-zeta. *Gene* **132**: 305–306

Bischoff FR and Ponstingl H (1991) Catalysis of guanine nucleotide exchange on Ran by the mitotic regulator RCC1. *Nature* **354**: 80–82

Brannan CI, Dees EC, Ingram RS and Tilghman SM (1990) The product of the H19 gene may function as an RNA. *Mol Cell Biol* **19**: 28–36

Crackower MA, Scherer SW, Rommens JM, Hui CC, Poorkaj P, Soder S, Cobben JM, Hudgins L, Evans JP and Tsui LC (1996) Characterization of the split hand/split foot malformationlocus SHFM1 at 7q21.3–q22.1 and analysis of a candidate gene for its expression during limb development. *Hum Mol Gen* **5**: 571–579

Deblandre GA, Marinx OP, Evans SS, Majaj S, Leo O, Caput D, Huez GA and Wathelet MG (1995) Expression cloning of an interferon-inducible 17-kDa membrane protein implicate in the control of cell growth. *J Biol Chem* **270**: 23860–23866

De La Salmonière P, Grob J-J, Dreno B, Delaunay M and Chastang C (2000) White blood cell count: a prognostic factor and possible subset indicator of optimal treatment with low dose adjuvant interferon in primary melanoma. *Clin Cancer Res* **6**: 4713–4718

Der SD, Zhou A, Williams BR and Silverman RH (1998) Identification of genes differentially regulated by interferon alpha, beta, or gamma using oligonucleotide arrays. *Proc Natl Acad Sci USA* **95**: 15623–15628

Fambrough D, McClure K, Kazlauskas A and Lander ES (1999) Diverse signaling pathways activated by growth factor receptors induce broadly overlapping, rather than independent, sets of genes. *Cell* **97**: 727–741

Grob JJ, Dreno B, de la Salmonière P, Delaunay M, Cupissol D, Guillot B, Souteyrand P, Sassolas B, Cesarini J-P, Lionnet S, Lok C, Chastang C and Bonerandi JJ (1998) Randomised trial of interferon α-2a as adjuvant therapy in resected primary melanoma thicker than 1.5 mm without clinically detectable node metastases. *Lancet* **351**: 1905–1910

Gurney ME, Heinrich SP, Lee MR and Yin HS (1986) Molecular cloning and expression of neuroleukin, a neurotrophic factor for spinal and sensory neurons. *Science* **234**: 566–574

Itoh S, Harada H, Fujita T, Mimura T and Taniguchi T (1989) Sequence of a cDNA coding for human IRF-2. *Nucleic Acids Res* **17**: 8372–8372

Iyer VR, Eisen MB, Ross DT, Schuler G, Moore T, Lee JCF, Trent JM, Staudt LM, Hudson J, Boguski MS, Lashkari D, Shalon D, Botstein D and Brown PO (1999) The transcriptional program in the response of human fibroblasts to serum. *Science* **283**: 83–87

Keilholz U and Eggermont AMM (2000) The emerging role of cytokines in the treatment of advanced melanoma. *Oncology* **58**: 89–95

Kirkwood JM (1998) Adjuvant IFN alpha2 therapy of melanoma. *Lancet* **351**: 1901–1903

Lipshutz RJ, Fodor SPA, Gingeras TR and Lockhart DJ (1999) High density synthetic oligonucleotide arrays. *Nature Genet* **21**: 20–24

Lüscher U, Filgueira L, Juretic A, Zuber M, Lüscher NJ, Heberer M and Spagnoli GC (1994) The pattern of cytokine gene expression in freshly excised human metastatic melanoma suggests a state of reversible anergy of tumor-infiltrating lymphocytes. *Int J Cancer* **57**: 612–619

Luster AD, Weinshank RL, Feinman R and Ravetch JV (1988) Molecular and biochemical characterization of a novel gamma-interferon-inducible protein. *J Biol Chem* **263**: 12036–12043

Lutfalla G, Holland SJ, Cinato E, Monneron D, Reboul J, Rogers NC, Smith JM, Stark GR, Gardiner K, Mogensen KE, Kerr IM and Uze G (1995) Mutant U5A cells are complemented by an interferon-alpha beta receptor subunit generated by alternative processing of a new member of a cytokine receptor gene cluster. *EMBO J* **14**: 5100–5108

Mahadevappa M and Warrington JA (1999) A high-density probe array sample preparation method using 10-to 100-fold fewer cells. *Nat Biotechnol* **17**: 1134–1136

Marton MJ, DeRisi JL, Bennett HA, Iyer VR, Meyer MR, Roberts CJ, Stoughton R, Burchard J, Slade D, Dai H, Bassett DE, Hartwell LH, Brown PO and Friend SH (1998) Drug target validation and identification of secondary drug target effects using DNA microarrays. *Nature Med* **4**: 1293–1301

Nigro CL, Venesio T, Reymond A, Meroni G, Alberici P, Cainarca S, Enrico F, Stack M, Ledbetter DH, Liscia DS, Ballabio A and Carrozzo R (1998) The human ROX gene genomic structure and mutation analysis in human breast tumors. *Genomics* **49**: 275–282

Noppen C, Lévy F, Burri L, Zajac P, Remmel E, Schaefer C, Lüscher U, Heberer M and Spagnoli GC (2000) Naturally processed and concealed HLA-A2.1 restricted epitopes from tumor associated antigen tyrosinase-related protein-2. *Int J Cancer* **87**: 241–246

Pansky A, Hildebrand P, Fasler-Kann E, Baselgia L, Ketterer S, Beglinger C and Heim MH (2000) Defective Jak-STAT signal transduction pathway in melanoma cells resistant to growth inhibition by interferon-α. *Int J Cancer* **85**: 720–725

Ralph SJ, Wines BD, Payne MJ, Grubb D, Hatzinisiriou I, Linnane AW and Devenish RJ (1995) Resistance of melanoma cell lines to interferons correlates with reduction of IFN-induced tyrosine phosphorilation. Induction of the anti-viral state by IFN is prevented by tyrosine kinase inhibitors. *J Immunol* **154**: 2248–2256

Rasmussen UB, Wolf C, Mattei MG, Chenard MP, Bellocq JP, Chambon P, Rio MC and Basset P (1993) Identification of a new interferon-alpha-inducible gene (p27) on human chromosome 14q32 and its expression in breast carcinoma. *Cancer Res* **53**: 4096–4101

Rogge L, Bianchi E, Biffi M, Bono E, Chang S-YP, Alexander H, Santini C, Ferrari G, Sinigaglia L, Seiler M, Neeb M, Mous J, Sinigaglia F and Certa U (2000) Transcripts imaging of the development of human T helper cells using oligonucleotide arrays. *Nature Genet* **25**: 96–101

Schuurig E (1995) The involvement of the chromosome 11q13 region in human malignancies: cyclin D1 and EMS1 are two new candidate oncogenes – a review. *Gene* **59**: 83–96

Spagnoli GC, Schaefer C, Willimann TE, Kocher T, Amoroso A, Juretic A, Zuber M, Lüscher U, Harder F and Heberer M (1995) Peptide specific CTL in tumor-infiltrated lymphocytes from metastatic melanomas expressing MART-1/Melan-A, gp100 and tyrosinase genes: a study in an unselected group of HLA-A2.1-positive patients. *Int J Cancer* **64**: 309–315

Tenhunen J, Salminen M, Lundstrom K, Kiviluoto T, Savolainen R and Ulmanen I (1994) Genomic organization of the human cathecol O-methyltransferase gene and its expression from two distinct promoters. *Eur J Biochem* **223**: 1049–1059

Trapani JA, Dawson M, Apostolidis VA and Browne KA (1994) Genomic organization of IF1I6, an interferon-inducible gene whose expression is associated with human myeloid cell differentiation: correlation of predicted protein domains with exon organization. *Immunogenetics* **40**: 415–424

Vial T and Descotes J (1994) Clinical toxicity of Interferons. *Drug Safety* **10**: 115–150

Welsh M, Mares J, Karlsson T, Lavergne C, Breant B and Claesson-Welsh L (1994) Shb is a ubiquitously expressed Src homology 2 protein. *Oncogene* **9**: 19–27

Wong LH, Krauer KG, Hatzinisiriou I, Estcourt MJ, Hersey P, Tam ND, Edmonson S, Devenish RJ and Ralph SJ (1997) Interferon-resistant human melanoma cells are deficient in ISGF3 components, STAT1, STAT2, and p48-ISGF3 gamma. *J Biol Chem* **272**: 28779–28785

 © 2001 Cancer Research Campaign

## References

[PDF_00761] Acampora D., D'Esposito M., Faiella A., Pannese M., Migliaccio E., Morelli F., Stornaiuolo A., Nigro V., Simeone A., Boncinelli E. (1989). The human HOX gene family.. Nucleic Acids Res.

[PDF_00764] Adams F., Fernandez F., Mavligit G. (1988). Interferon-induced organic mental disorders associated with unsuspected pre-existing neurologic abnormalities.. J Neurooncol.

[PDF_00767] Aebi M., Fäh J., Hurt N., Samuel C. E., Thomis D., Bazzigher L., Pavlovic J., Haller O., Staeheli P. (1989). cDNA structures and regulation of two interferon-induced human Mx proteins.. Mol Cell Biol.

[PDF_00770] Agarwala S. S., Kirkwood J. M. (1996). Interferons in melanoma.. Curr Opin Oncol.

[PDF_00772] Barbee J. L., Deutscher S. L., Loomis C. R., Burns D. J. (1993). The cDNA sequence encoding human protein kinase C-zeta.. Gene.

[PDF_00774] Bischoff F. R., Ponstingl H. (1991). Catalysis of guanine nucleotide exchange on Ran by the mitotic regulator RCC1.. Nature.

[PDF_00776] Brannan C. I., Dees E. C., Ingram R. S., Tilghman S. M. (1990). The product of the H19 gene may function as an RNA.. Mol Cell Biol.

[PDF_00778] Crackower M. A., Scherer S. W., Rommens J. M., Hui C. C., Poorkaj P., Soder S., Cobben J. M., Hudgins L., Evans J. P., Tsui L. C. (1996). Characterization of the split hand/split foot malformation locus SHFM1 at 7q21.3-q22.1 and analysis of a candidate gene for its expression during limb development.. Hum Mol Genet.

[PDF_00783] Deblandre G. A., Marinx O. P., Evans S. S., Majjaj S., Leo O., Caput D., Huez G. A., Wathelet M. G. (1995). Expression cloning of an interferon-inducible 17-kDa membrane protein implicated in the control of cell growth.. J Biol Chem.

[PDF_00791] Der S. D., Zhou A., Williams B. R., Silverman R. H. (1998). Identification of genes differentially regulated by interferon alpha, beta, or gamma using oligonucleotide arrays.. Proc Natl Acad Sci U S A.

[PDF_00794] Fambrough D., McClure K., Kazlauskas A., Lander E. S. (1999). Diverse signaling pathways activated by growth factor receptors induce broadly overlapping, rather than independent, sets of genes.. Cell.

[PDF_00797] Grob J. J., Dreno B., de la Salmonière P., Delaunay M., Cupissol D., Guillot B., Souteyrand P., Sassolas B., Cesarini J. P., Lionnet S. (1998). Randomised trial of interferon alpha-2a as adjuvant therapy in resected primary melanoma thicker than 1.5 mm without clinically detectable node metastases. French Cooperative Group on Melanoma.. Lancet.

[PDF_00802] Gurney M. E., Heinrich S. P., Lee M. R., Yin H. S. (1986). Molecular cloning and expression of neuroleukin, a neurotrophic factor for spinal and sensory neurons.. Science.

[PDF_00805] Itoh S., Harada H., Fujita T., Mimura T., Taniguchi T. (1989). Sequence of a cDNA coding for human IRF-2.. Nucleic Acids Res.

[PDF_00807] Iyer V. R., Eisen M. B., Ross D. T., Schuler G., Moore T., Lee J. C., Trent J. M., Staudt L. M., Hudson J., Boguski M. S. (1999). The transcriptional program in the response of human fibroblasts to serum.. Science.

[PDF_00811] Keilholz U., Eggermont A. M. (2000). The emerging role of cytokines in the treatment of advanced melanoma. For the EORTC Melanoma Cooperative Group.. Oncology.

[PDF_00813] Kirkwood J. M. (1998). Adjuvant IFN alpha2 therapy of melanoma.. Lancet.

[PDF_00815] Lipshutz R. J., Fodor S. P., Gingeras T. R., Lockhart D. J. (1999). High density synthetic oligonucleotide arrays.. Nat Genet.

[PDF_00836] Lo Nigro C., Venesio T., Reymond A., Meroni G., Alberici P., Cainarca S., Enrico F., Stack M., Ledbetter D. H., Liscia D. S. (1998). The human ROX gene: genomic structure and mutation analysis in human breast tumors.. Genomics.

[PDF_00821] Luster A. D., Weinshank R. L., Feinman R., Ravetch J. V. (1988). Molecular and biochemical characterization of a novel gamma-interferon-inducible protein.. J Biol Chem.

[PDF_00824] Lutfalla G., Holland S. J., Cinato E., Monneron D., Reboul J., Rogers N. C., Smith J. M., Stark G. R., Gardiner K., Mogensen K. E. (1995). Mutant U5A cells are complemented by an interferon-alpha beta receptor subunit generated by alternative processing of a new member of a cytokine receptor gene cluster.. EMBO J.

[PDF_00817] Lüscher U., Filgueira L., Juretic A., Zuber M., Lüscher N. J., Heberer M., Spagnoli G. C. (1994). The pattern of cytokine gene expression in freshly excised human metastatic melanoma suggests a state of reversible anergy of tumor-infiltrating lymphocytes.. Int J Cancer.

[PDF_00829] Mahadevappa M., Warrington J. A. (1999). A high-density probe array sample preparation method using 10- to 100-fold fewer cells.. Nat Biotechnol.

[PDF_00832] Marton M. J., DeRisi J. L., Bennett H. A., Iyer V. R., Meyer M. R., Roberts C. J., Stoughton R., Burchard J., Slade D., Dai H. (1998). Drug target validation and identification of secondary drug target effects using DNA microarrays.. Nat Med.

[PDF_00840] Noppen C., Lévy F., Burri L., Zajac P., Remmel E., Schaefer C., Lüscher U., Heberer M., Spagnoli G. C. (2000). Naturally processed and concealed HLA-A2.1-restricted epitopes from tumor-associated antigen tyrosinase-related protein-2.. Int J Cancer.

[PDF_00844] Pansky A., Hildebrand P., Fasler-Kan E., Baselgia L., Ketterer S., Beglinger C., Heim M. H. (2000). Defective Jak-STAT signal transduction pathway in melanoma cells resistant to growth inhibition by interferon-alpha.. Int J Cancer.

[PDF_00847] Ralph S. J., Wines B. D., Payne M. J., Grubb D., Hatzinisiriou I., Linnane A. W., Devenish R. J. (1995). Resistance of melanoma cell lines to interferons correlates with reduction of IFN-induced tyrosine phosphorylation. Induction of the anti-viral state by IFN is prevented by tyrosine kinase inhibitors.. J Immunol.

[PDF_00851] Rasmussen U. B., Wolf C., Mattei M. G., Chenard M. P., Bellocq J. P., Chambon P., Rio M. C., Basset P. (1993). Identification of a new interferon-alpha-inducible gene (p27) on human chromosome 14q32 and its expression in breast carcinoma.. Cancer Res.

[PDF_00855] Rogge L., Bianchi E., Biffi M., Bono E., Chang S. Y., Alexander H., Santini C., Ferrari G., Sinigaglia L., Seiler M. (2000). Transcript imaging of the development of human T helper cells using oligonucleotide arrays.. Nat Genet.

[PDF_00862] Spagnoli G. C., Schaefer C., Willimann T. E., Kocher T., Amoroso A., Juretic A., Zuber M., Luscher U., Harder F., Heberer M. (1995). Peptide-specific CTL in tumor infiltrating lymphocytes from metastatic melanomas expressing MART-1/Melan-A, gp100 and Tyrosinase genes: a study in an unselected group of HLA-A2.1-positive patients.. Int J Cancer.

[PDF_00867] Tenhunen J., Salminen M., Lundström K., Kiviluoto T., Savolainen R., Ulmanen I. (1994). Genomic organization of the human catechol O-methyltransferase gene and its expression from two distinct promoters.. Eur J Biochem.

[PDF_00871] Trapani J. A., Dawson M., Apostolidis V. A., Browne K. A. (1994). Genomic organization of IFI16, an interferon-inducible gene whose expression is associated with human myeloid cell differentiation: correlation of predicted protein domains with exon organization.. Immunogenetics.

[PDF_00875] Vial T., Descotes J. (1994). Clinical toxicity of the interferons.. Drug Saf.

[PDF_00877] Welsh M., Mares J., Karlsson T., Lavergne C., Bréant B., Claesson-Welsh L. (1994). Shb is a ubiquitously expressed Src homology 2 protein.. Oncogene.

[PDF_00879] Wong L. H., Krauer K. G., Hatzinisiriou I., Estcourt M. J., Hersey P., Tam N. D., Edmondson S., Devenish R. J., Ralph S. J. (1997). Interferon-resistant human melanoma cells are deficient in ISGF3 components, STAT1, STAT2, and p48-ISGF3gamma.. J Biol Chem.

[PDF_00787] de La Salmonière P., Grob J. J., Dreno B., Delaunay M., Chastang C. (2000). White blood cell count: a prognostic factor and possible subset indicator of optimal treatment with low-dose adjuvant interferon in primary melanoma.. Clin Cancer Res.

